# Food Insecurity Among Medicare Beneficiaries in 2017–2022: A Longitudinal Cohort Study

**DOI:** 10.1111/jgs.70441

**Published:** 2026-04-15

**Authors:** David D. Kim, Charles J. Duncan, Elliott Crummer

**Affiliations:** ^1^ Department of Medicine, Biological Sciences Division University of Chicago Chicago Illinois USA; ^2^ Department of Public Health Sciences, Biological Sciences Division University of Chicago Chicago Illinois USA; ^3^ Graduate Program in Health Administration and Policy University of Chicago Chicago Illinois USA; ^4^ Department of Economics University of California Los Angeles California USA

**Keywords:** food insecurity, Medicare, social determinants of health

## Abstract

**Background:**

Food insecurity affects many older adults and is associated with a range of adverse health outcomes. However, most prior research has relied on cross‐sectional data, and evidence on predictors of subsequent food insecurity among Medicare beneficiaries remains limited.

**Methods:**

We conducted a retrospective longitudinal cohort study of 12,029 individuals using 2017–2022 Medicare Current Beneficiary Survey data. We applied linear lagged dependent variable models to estimate predicted probabilities of food insecurity in the subsequent year, based on the prior year's demographics, socioeconomic characteristics, health conditions, and food insecurity status. Food insecurity was assessed using the USDA's 6‐item short‐form survey. Demographic and socioeconomic characteristics were measured from self‐reported survey responses, and health conditions were identified using medical claims data.

**Results:**

In adjusted linear probability models, the strongest predictor of subsequent‐year food insecurity was prior‐year food insecurity, which is associated with a 43 percentage point (p.p.) higher probability of food insecurity in the subsequent year (95% CI: 38.7–47.2). Other important predictors included self‐reported “poor” general health (6.5 p.p. [0.5–12.4]), inability to afford dental care (6.4 p.p [3.1–9.7]), difficulty paying medical bills (5.2 p.p. [1.5–8.9]), not filling prescriptions due to costs (4.4 p.p. [2.2–6.5]), and Hispanic ethnicity (4.1 p.p. [1.7–6.6]).

**Conclusions:**

Among older Medicare beneficiaries, prior food insecurity and markers of acute financial strain, including difficulty affording dental care or prescriptions, strongly predict future food insecurity, even after adjusting for their income. Incorporating these indicators into screening efforts may improve identification of beneficiaries at risk of food insecurity. Furthermore, linking screening efforts with navigation and benefits enrollment support, alongside broader policy reforms to reduce financial strain, may help mitigate food insecurity and its downstream health consequences in this population.

## Summary

- Key points
  ○ Food insecurity is highly persistent among older adults, and prior‐year food insecurity is the strongest predictor of subsequent‐year food insecurity.
  ○ Acute healthcare‐related financial strain, such as difficulty affording dental care or prescriptions, strongly predicts future food insecurity even after adjusting for income.
  ○ Black‐White racial‐ethnic disparities are largely explained by underlying socioeconomic factors, whereas Hispanic ethnicity remains independently associated with food insecurity after covariate adjustment, suggesting the presence of additional structural or access‐related drivers not captured by observed covariates.
- Why does this paper matter?
  ○ This study is the first longitudinal evidence on predictors of food insecurity among older US adults, highlighting healthcare‐related financial burdens as key indicators of future food insecurity. Incorporating these indicators into screening efforts may improve identification of individuals at risk of food insecurity and help guide targeted interventions to reduce food insecurity among older adults.

## 1 Introduction

Food insecurity, defined by the US Department of Agriculture as a “household‐level economic and social condition of limited or uncertain access to adequate food,” affects more than 10% of American households [1]. The lingering effects of the COVID‐19 pandemic, increased food prices, and the decisions to end Supplemental Nutrition Assistance Program (SNAP) Emergency Allotments early further worsened the problem [2, 3].

The associations between food insecurity and adverse health consequences have been well documented. Individuals who are food insecure are more likely to have diabetes and poor glycemic control [4, 5], hypertension, hyperlipidemia, atherosclerotic cardiovascular disease [6, 7], obesity [8], and worse oral health [9], among other conditions [10]. Food insecurity is also associated with worse mental health, increasing the risk of anxiety, stress, and depression [11, 12], and additional healthcare utilization and spending [13, 14].

In particular, food insecurity poses a unique challenge to older adults, who encounter more significant disease burden, disability, low incomes, and social isolation that can exacerbate the harm caused by inadequate intakes of energy and essential micronutrients [15]. Older adults with food insecurity are more likely to report poor or fair health, experience limitations in activities of daily living, and live with congestive heart failure, gum disease, and asthma [16]. Although adults aged 65 and over had a slightly lower rate of food insecurity (9.3%) than the national average (13.5%) in 2023, the relatively persistent rate of food insecurity among older adults is concerning [1, 17].

Prior work has documented that those in racial‐ethnic minority and younger age groups and with limited economic resources, lower educational attainment, unmarried status, and disablility are all associated with an increased risk of food insecurity [18, 19]. However, most of these studies focus on children and healthy working‐age adults, limiting our understanding of food insecurity among older adults [20]. In addition, many studies have examined the consequences of food insecurity rather than factors that predicit food insecurity, and those studies that do attempt such investigations often use cross‐sectional designs, making it difficult to assess reverse causality [21, 22]. Using longitudinal data of Medicare beneficiaries, our study aims to examine key factors predicting food insecurity among older adults.

## 2 Methods

### 2.1 Data and Study Sample

We analyzed the 2017–2022 Medicare Current Beneficiary Survey (MCBS) Limited Data Set, a continuous, in‐person, nationally representative survey of the Medicare population. The MCBS contains individual‐level data on demographics, access to medical care, healthcare utilization, socioeconomics, and underlying health conditions. We constructed five panels of the study sample consisting of individuals aged 65+ who completed the MCBS's food security module in any two consecutive years from 2017–2018 to 2021–2022. We excluded individuals living in facilities or nursing homes due to limited information. This study was exempted from IRB review because the data contain no specific direct identifiers.

### 2.2 Measures

The primary outcome for all analyses was a binary indicator of food insecurity, defined as respondingas affirmative – (1) “sometimes”/“often” on HH3 and HH4, (2) yes on AD1, AD2, and AD3, and (3) almost every month/some months on AD1a – to at least two items on the USDA's Six‐Item Short Form for food insecurity [23], available in the MCBS food security module. Subsequent‐year food insecurity was modeled as the dependent variable, with prior‐year food insecurity as an independent variable.

Consistent with prior studies [24, 25, 26], we measured independent variables in the domains of demographics, socioeconomic characteristics, and health conditions. Demographics included age, sex (male, female), self‐reported race and ethnicity (White or other, Black, Hispanic), marital status (yes, no), and household size. Socioeconomic characteristics captured multiple dimensions of financial resources relevant to Medicare beneficiaries. Binary indicators included income‐to‐poverty ratio ≤ 200%, home ownership, employment, less than high school education, and 401(k) ownership. We also included four measures of healthcare‐related financial strain: (1) having trouble getting healthcare due to cost, (2) being unable to get dental care due to cost, (3) having trouble paying medical bills, and (4) often or sometimes not filling prescriptions due to costs (i.e., cost‐related prescription non‐adherence). All demographic and socioeconomic characteristics were constructed based on MCBS survey responses.

Health conditions were identified from MCBS data based on medical claims that had been filed during the survey year, rather than self‐report. Cardiovascular disease included acute myocardial infarction (heart attack), atrial fibrillation, ischemic heart disease, stroke/transient ischemic attack, and congestive heart failure based on clinical guidelines [27]. Depression and anxiety were defined using the Patient Health Questionnaire‐9 (PHQ‐9) (≥ 15) and Generalized Anxiety Disorder 2‐item (GAD‐2) (≥ 3) cutoffs, respectively [28, 29, 30]. Other conditions were defined directly from MCBS data.

### 2.3 Statistical Analysis

To examine associations between these domains (demographics, socioeconomic characteristics, and health conditions) and food insecurity among Medicare beneficiaries, we constructed three nested models with sequential adjustment. Model 1 included demographics only, Model 2 additionally adjusted for socioeconomic factors, and Model 3 further included health conditions. To reduce bias from reverse causation, we employed a lagged dependent variable model to associate covariates in an index year (e.g., 2017) with food insecurity in the subsequent year (e.g., 2018), following prior approaches [31, 32]. This approach leverages temporal ordering and accounts for persistence in food insecurity over time [33]. To choose the best‐fitting models for a binary outcome, we compared three functional forms: a linear probability model (LPM), a logit model, and a probit model. Among the three models, we chose the LPM because of its performance based on Akaike information criteria, Bayesian information criteria, and log‐likelihood goodness‐of‐fit tests (Table S1) [34].

The primary outputs were predicted probabilities of subsequent‐year food insecurity, adjusted for prior‐year food insecurity and relevant covariates from each domain. The base case analysis pooled Medicare beneficiaries aged 65+ from all five panels.

To examine whether predictors of food insecurity differed across key demographic groups that are commonly highlighted in food insecurity disparities research, we conducted subgroup analyses by sex (male, female), race and ethnicity (White or other, Black, Hispanic), age (65–74, 75+), and baseline food insecurity status (food secure, food insecure in first observed year). Secondary analyses estimated panel‐specific effects to assess whether associations varied over time, including during the COVID‐19 period. Analyses were performed in R version 4.4.1 (June 2025). Following MCBS analytic guidance, survey weights were applied to produce nationally representative estimates and account for non‐response in the food insecurity questionnaire [35].

## 3 Results

### 3.1 Sample Characteristics

We included 12,029 respondents in the analytic sample, representing 96,373,115 Medicare beneficiaries from 2017 to 2022. Sample characteristics and subgroup‐specific food insecurity prevalence are shown in Table 1. Overall, 7.1% (95% CI: 6.4–7.8) of beneficiaries experienced food insecurity, with substantial differences across population subgroups. Among those food insecure in a prior year, 55.3% (52.0–58.7) remained food insecure in the following year. Food insecurity prevalence was higher among females (8.2% [7.5–8.9]) than males (5.7% [4.9–6.5]), and higher among Black (17.8% [15.1–20.5]) beneficiaries and Hispanic (18.9% [16.2–21.6]) beneficiaries than White beneficiaries (4.8% [4.3–5.3]).

**TABLE 1:** Sample characteristics of Medicare beneficiaries aged 65+, 2017–2022 (12,029 individuals representing 96,373,115).

Characteristic	Survey‐weighted proportion (%)	Food insecurity (%) mean (95% CI)
Total population		7.1% (6.4, 7.8)
Food insecurity in the prior year	7.0%	55.3% (52.0, 58.7)
Demographics		
Aged 65–69	31.5%	8.7% (7.6, 9.9)
Aged 70–74	28.2%	7.2% (6.1, 8.3)
Aged 75–79	19.2%	6.4% (5.4, 7.3)
Aged 80+	21.0%	5.1% (4.5, 5.8)
Male	43.9%	5.7% (4.9, 6.5)
Female	56.1%	8.2% (7.5, 8.9)
White	78.0%	4.8% (4.3, 5.3)
Black	9.1%	17.8% (15.1, 20.5)
Hispanic	7.0%	18.9% (16.2, 21.6)
Asian	2.0%	9.3% (5.3, 13.2)
Native American	0.4%	18.8% (9.5, 28.0)
Other race	0.9%	8.6% (3.0, 14.3)
Unmarried	44.1%	10.9% (9.9, 11.9%)
Married	55.9%	4.1% (3.5, 4.6)
Socioeconomic characteristics		
Income‐to‐poverty ratio ≤ 200%	31.3%	17.8% (16.4, 19.2)
Does not own home	16.2%	18.2% (16.3, 20.1)
Owns home	83.8%	4.5% (4.0, 5.1)
Unemployed	81.2%	7.9% (7.2, 8.6)
Employed	18.8%	3.6% (2.8, 4.3)
Less than high school education	11.2%	19.6% (17.2, 22.0)
Had a problem paying medical bills	5.3%	35.0% (31.9, 38.1)
Enrolled in Medicare Advantage	41.0%	9.8% (8.8, 10.7)
Having trouble getting health care due to cost	0.5%	35.3% (27.9, 42.8)
Unable to get dental care due to cost	4.0%	31.2% (27.9, 34.5)
Often/sometimes does not fill Rx due to cost	7.3%	21.1% (18.2, 24.0)
Has 401(k)	46.1%	1.7% (1.3, 2.1)
Health conditions		
General health felt to be poor	2.4%	22.7% (18.1, 27.3)
Has difficulty walking	21.2%	15.6% (14.3, 16.9)
Has cognitive difficulties	9.9% (9.3%, 10.6%)	17.1% (14.7, 19.6)
Has at least moderately severe depression	1.5%	34.3% (28.6, 40.1)
Has clinical anxiety	7.5%	19.9% (17.3, 22.5)
Limited social activities due to health	20.3%	15.5% (13.9, 17.1)
Has smoked 100+ cigarettes	50.0%	7.8% (6.9, 8.6)
Has Alzheimer's	0.9%	8.3% (3.4, 13.1)
Has CKD	12.7%	9.4% (7.9, 10.9)
Has COPD	5.1%	11.7% (9.1, 14.3)
Has cardiovascular disease	19.1% (18.1%, 20.2%)	7.8% (6.5, 9.1)
Has diabetes	15.2%	10.9% (9.2, 12.6)
Has rheumatoid/osteoarthritis	19.2%	6.5% (5.6, 7.5)

Abbreviations: CKD = chronic kidney disease; COPD = chronic obstructive pulmonary disease.

Beneficiaries reporting healthcare‐related financial strain had a much higher prevalence, including those struggling to afford health care (35.3% [27.9–42.8]), having trouble paying medical bills (35.0% [31.9–38.1]), and unable to afford dental care (31.2% [27.0–34.5]). Food insecurity prevalence was also elevated among beneficiaries with underlying health conditions.

### 3.2 Key Factors Associated With Food Insecurity in Older Adults

Prior‐year food insecurity was the strongest predictor of subsequent‐year food insecurity among Medicare beneficiaries aged 65+ (Table 2). After adjusting for demographic, socioeconomic, and health characteristics, prior‐year food insecurity was associated with a 43.0 percentage point (p.p) higher probability of food insecurity in the subsequent year (95% CI: 38.7–47.2). Healthcare‐related financial strain was also strongly associated with food insecurity, including foregoing dental care due to cost (6.4 p.p. [3.1–9.7]), difficulty paying medical bills (5.2 p.p. [1.5–8.9]), cost‐related prescription non‐adherence (4.4 p.p. [2.2–6.5]), and income below 200% federal poverty level (3.9 p.p. [2.8–5.0]). Although specific health conditions were not significant predictors by themselves, broader health indicators were associated with higher risk, including self‐reported poor health (6.5 p.p. [0.5–12.4]) and health‐related limitations in social activities (3.1 p.p. [1.5–4.6]). Protective factors against food insecurity included being married (−1.6 p.p. [−2.6 to −0.6]) and having a 401 k (−1.1 p.p. [−1.9 to −0.3]). After adjustment, female sex, Black race, and household size were not statistically significant. Results from non‐linear models are reported in Table S1.

**TABLE 2:** Linear probability models of food insecurity among Medicare beneficiaries aged 65+, 2017–2022.

	Model 1 (demographic)	Model 2 (demographic + socioeconomic)	Model 3 (demographic + socioeconomic + health)
Food insecurity in the prior year	0.503*** **(0.468, 0.539)**	0.433*** **(0.393, 0.474)**	0.430*** **(0.387, 0.472)**
Demographics			
Age	−0.001*** **(−0.002, −0.001)**	−0.001*** **(−0.002, −0.001)**	−0.002*** **(−0.002, −0.001)**
Female [vs. Male]	0.008* **(0.000, 0.016)**	0.004 (−0.004, 0.012)	0.002 (−0.006, 0.010)
Black [vs. White/Other]	0.049*** **(0.029, 0.070)**	0.019 (−0.004, 0.042)	0.018 (−0.005, 0.041)
Hispanic [vs. White/Other]	0.054*** **(0.032, 0.075)**	0.035** **(0.011, 0.059)**	0.041** **(0.017, 0.066)**
Married [vs. Not married]	−0.036*** **(−0.044, −0.027)**	−0.019*** **(−0.028, −0.009)**	−0.016** **(−0.026, −0.006)**
Household size	0.006** **(0.002, 0.011)**	0.003 (−0.002, 0.009)	0.002 (−0.004, 0.008)
Socioeconomic characteristics			
Income‐poverty ratio ≤ 200%	—	0.044*** **(0.033, 0.055)**	0.039*** **(0.028, 0.050)**
Owns home	—	−0.020* **(−0.036, −0.004)**	−0.016 (−0.032, 0.000)
Employed	—	−0.005 (−0.014, 0.004)	0.002 (−0.007, 0.011)
Less than high school education	—	0.004 (−0.019, 0.027)	−0.003 (−0.027, 0.021)
Had a problem paying medical bills	—	0.059** **(0.024, 0.095)**	0.052** **(0.015, 0.089)**
Enrolled in Medicare Advantage	—	−0.002 (−0.010, 0.006)	0.007 (−0.002, 0.015)
Having trouble getting health care due to cost	—	−0.032 (−0.135, 0.071)	−0.047 (−0.16, 0.065)
Unable to get dental care due to cost	—	0.070*** **(0.038, 0.103)**	0.064*** **(0.031, 0.097)**
Often/sometimes does not fill Rx due to cost	—	0.056*** **(0.036, 0.076)**	0.044*** **(0.022, 0.065)**
Has 401 k	—	−0.012** **(−0.02, −0.005)**	−0.011** **(−0.019, −0.003)**
Health conditions			
General health felt to be poor	—	—	0.065* **(0.005, 0.124)**
Has difficulty walking	—	—	0.009 (−0.004, 0.022)
Has cognitive difficulties	—	—	0.001 (−0.025, 0.027)
Has at least moderately severe depression	—	—	0.000 (−0.061, 0.061)
Has clinical anxiety	—	—	0.000 (−0.018, 0.019)
Limited social activities due to health	—	—	0.031*** **(0.015, 0.046)**
Has smoked 100+ cigarettes	—	—	0.002 (−0.006, 0.010)
Has Alzheimer's/dementia	—	—	−0.027 (−0.057, 0.003)
Has CKD	—	—	0.010 (−0.006, 0.026)
Has COPD	—	—	−0.014 (−0.037, 0.009)
Has cardiovascular disease	—	—	0.003 (−0.010, 0.017)
Has diabetes	—	—	0.006 (−0.007, 0.020)
Has rheumatoid/osteoarthritis	—	—	0.008 (−0.002, 0.018)
*N* (observations)	16,412	14,970	13,769
*N* (individuals)	11,749	10,796	10,059

*Note*: Boldface indicates statistical significance (*p < 0.01, **p < 0.05, ***p < 0.001). 95% confidence intervals in parentheses.

Abbreviations: CKD = chronic kidney disease; COPD = chronic obstructive pulmonary disease.

### 3.3 Subgroup Analysis

Sex‐stratified analyses (Table 3) showed few differences from the overall sample. Prior‐year food insecurity was more predictive among female beneficiaries (45.1 p.p. [39.5–50.6]) than male beneficiaries (38.9 p.p. [31.9–45.9]). Hispanic ethnicity was not a significant predictor among males but remained significant among females (7.3 p.p. [3.4–11.2]). Race and ethnicity‐stratified analyses (Table 4) suggested that prior‐year food insecurity was a stronger predictor among White beneficiaries (46.2 p.p. [40.5–51.9]) than for Black (37.9 p.p. [29.3–46.5]) and Hispanic (36.8 p.p. [26.3–47.3]) beneficiaries, indicating greater persistence of food insecurity among White beneficiaries and more transitions between food security states among other groups.

**TABLE 3:** Linear probability models of food insecurity among Medicare beneficiaries aged 65+, 2017–2022, stratified by sex.

	Male	Female
Food insecurity in the prior year	0.389*** **(0.319, 0.459)**	0.451*** **(0.395, 0.506)**
Demographics		
Age	−0.001** **(−0.002, 0.000)**	−0.002*** **(−0.002, −0.001)**
Black [vs. White or other]	0.019 (−0.012, 0.050)	0.017 (−0.020, 0.054)
Hispanic [vs. White or other]	−0.001 (−0.033, 0.030)	0.073*** **(0.034, 0.112)**
Married [vs. Not married]	−0.015* **(−0.030, 0.000)**	−0.018* **(−0.033, −0.002)**
Household size	0.002 (−0.006, 0.010)	0.002 (−0.006, 0.011)
Income‐poverty ratio ≤ 200%	0.054*** **(0.034, 0.073)**	0.029*** **(0.015, 0.044)**
Owns home	−0.006 (−0.028, 0.017)	−0.022* **(−0.044, −0.001)**
Employed	−0.003 (−0.014, 0.007)	0.006 (−0.009, 0.021)
Less than high school education	0.001 (−0.028, 0.030)	−0.006 (−0.038, 0.026)
Had a problem paying medical bills	0.057* **(0.001, 0.112)**	0.048 (−0.003, 0.100)
Enrolled in Medicare Advantage	0.004 (−0.007, 0.016)	0.010 (−0.002, 0.022)
Having trouble getting healthcare due to cost	−0.031 (−0.194, 0.131)	−0.053 (−0.223, 0.116)
Unable to get dental care due to cost	0.030 (−0.013, 0.073)	0.082** **(0.034, 0.129)**
Often/sometimes does not fill Rx due to cost	0.045* **(0.008, 0.083)**	0.043** **(0.017, 0.068)**
Has 401(k)	−0.011* **(−0.021, −0.002)**	−0.012* **(−0.022, −0.001)**
Health conditions		
General health felt to be poor	0.033 (−0.052, 0.118)	0.078* **(0.003, 0.154)**
Has difficulty walking	0.002 (−0.020, 0.023)	0.012 (−0.007, 0.031)
Has cognitive difficulties	0.021 (−0.020, 0.061)	−0.010 (−0.043, 0.022)
Has at least moderately severe depression	0.009 (−0.120, 0.137)	−0.006 (−0.079, 0.068)
Has clinical anxiety	0.016 (−0.017, 0.049)	−0.008 (−0.034, 0.019)
Limited social activities due to health	0.045*** **(0.023, 0.068)**	0.022 (0.000, 0.045)
Has smoked 100+ cigarettes	−0.006 (−0.016, 0.004)	0.008 (−0.005, 0.020)
Has Alzheimer's/dementia	−0.023 (−0.059, 0.013)	−0.029 (−0.08, 0.022)
Has CKD	0.007 (−0.015, 0.029)	0.014 (−0.005, 0.034)
Has COPD	−0.040* **(−0.070, −0.009)**	0.004 (−0.026, 0.033)
Has cardiovascular disease	0.007 (−0.009, 0.023)	−0.001 (−0.019, 0.018)
Has diabetes	0.013 (−0.004, 0.030)	0.004 (−0.016, 0.024)
Has rheumatoid/osteoarthritis	0.001 (−0.013, 0.015)	0.012 (−0.002, 0.027)
*N* (observations)	6065	7701
*N* (individuals)	4438	5619

*Note*: Boldface indicates statistical significance (*p < 0.01, **p < 0.05, ***p < 0.001). 95% confidence intervals in parentheses.

Abbreviations: CKD = chronic kidney disease; COPD = chronic obstructive pulmonary disease.

**TABLE 4:** Linear probability models of food insecurity among Medicare beneficiaries aged 65+, 2017–2022, stratified by race and ethnicity.

	Black	Hispanic	White
Food insecurity in the prior year	0.379*** **(0.293, 0.465)**	0.368*** **(0.263, 0.473)**	0.462*** **(0.405, 0.519)**
Demographics			
Age	−0.002 (−0.006, 0.001)	−0.005* **(−0.010, −0.001)**	−0.001*** **(−0.002, −0.001)**
Female [vs. Male]	−0.010 (−0.059, 0.039)	0.046 (−0.019, 0.111)	0.000 (−0.008, 0.007)
Married [vs. Not married]	−0.022 (−0.067, 0.023)	−0.071* **(−0.135, −0.006)**	−0.011* **(−0.020, −0.002)**
Household size	0.002 (−0.016, 0.021)	0.003 (−0.022, 0.028)	0.001 (−0.006, 0.007)
Socioeconomic characteristics			
Income‐poverty ratio ≤ 200%	0.054* **(0.014, 0.094)**	0.058 (−0.009, 0.125)	0.033*** **(0.020, 0.045)**
Owns home	−0.041 (−0.097, 0.015)	−0.031 (−0.106, 0.044)	−0.005 (−0.020, 0.010)
Employed	0.012 (−0.033, 0.057)	0.032 (−0.047, 0.111)	0 (−0.010, 0.010)
Less than high school education	−0.036 (−0.091, 0.018)	0.005 (−0.063, 0.073)	−0.001 (−0.028, 0.026)
Had a problem paying medical bills	0.000 (−0.091, 0.09)	0.076 (−0.071, 0.222)	0.066** **(0.024, 0.109)**
Enrolled in Medicare Advantage	0.054* **(0.003, 0.105)**	0.048 (−0.004, 0.101)	0.000 (−0.007, 0.008)
Having trouble getting healthcare due to cost	−0.164 (−0.545, 0.216)	−0.058 (−0.481, 0.365)	−0.025 (−0.155, 0.104)
Unable to get dental care due to cost	0.158* **(0.023, 0.293)**	0.118 (−0.011, 0.247)	0.036* **(0.003, 0.068)**
Often/sometimes does not fill Rx due to cost	0.064 (−0.039, 0.166)	0.074 (−0.016, 0.164)	0.037*** **(0.017, 0.058)**
Has 401 k	0.010 (−0.043, 0.062)	−0.049 (−0.107, 0.010)	−0.011** **(−0.018, −0.003)**
Health conditions			
General health felt to be poor	0.119 (−0.082, 0.320)	−0.025 (−0.184, 0.134)	0.074* **(0.004, 0.144)**
Has difficulty walking	−0.003 (−0.060, 0.055)	0.034 (−0.047, 0.114)	0.009 (−0.004, 0.022)
Has cognitive difficulties	0.040 (−0.073, 0.153)	−0.020 (−0.132, 0.091)	−0.008 (−0.033, 0.017)
Has at least moderately severe depression	−0.080 (−0.284, 0.124)	−0.102 (−0.287, 0.082)	0.050 (−0.019, 0.119)
Has clinical anxiety	0.054 (−0.066, 0.174)	0.021 (−0.075, 0.118)	−0.007 (−0.029, 0.015)
Limited social activities due to health	0.055 (−0.021, 0.132)	0.036 (−0.059, 0.130)	0.022** **(0.006, 0.038)**
Has smoked 100+ cigarettes	0.011 (−0.043, 0.065)	−0.022 (−0.077, 0.033)	0.004 (−0.003, 0.011)
Has Alzheimer's/dementia	−0.131 (−0.397, 0.134)	0.054 (−0.298, 0.406)	−0.017 (−0.038, 0.003)
Has CKD	−0.001 (−0.071, 0.069)	0.091 (−0.009, 0.190)	0.008 (−0.009, 0.024)
Has COPD	−0.099 (−0.219, 0.021)	−0.046 (−0.197, 0.106)	−0.006 (−0.031, 0.019)
Has cardiovascular disease	0.035 (−0.041, 0.112)	−0.052 (−0.142, 0.038)	0.003 (−0.010, 0.016)
Has diabetes	0.034 (−0.026, 0.095)	0.033 (−0.064, 0.130)	0.000 (−0.013, 0.014)
Has rheumatoid/osteoarthritis	0.028 (−0.038, 0.095)	0.077 (−0.011, 0.165)	0.003 (−0.006, 0.012)
*N* (observations)	856	1101	11,013
*N* (individuals)	751	821	8023

*Note*: Boldface indicates statistical significance (*p < 0.01, **p < 0.05, ***p < 0.001). 95% confidence intervals in parentheses.

Abbreviations: CKD = chronic kidney disease; COPD = chronic obstructive pulmonary disease.

Age‐stratified analyses (Table S2) indicated that prior‐year food insecurity was more predictive among beneficiaries aged 65–74 years (45.1 p.p. [39.7–50.5]) than among those aged 75+ (36.8 p.p. [31.0–42.7]). In contrast, some financial strain indicators, such as inability to afford dental care and difficulty paying medical bills, had stronger associations among older beneficiaries. Analyses stratified by baseline food insecurity status (i.e., in the first observed year) showed distinct patterns (Table S3). Among beneficiaries who were food secure at baseline, subsequent food insecurity was associated with Black and Hispanic race and ethnicity and broader measures of socioeconomic vulnerability. In contrast, among those already food insecure, persistence was largely driven by the inability to afford dental care and prescription drugs.

### 3.4 Panel‐Specific Effects

**FIGURE 1:**
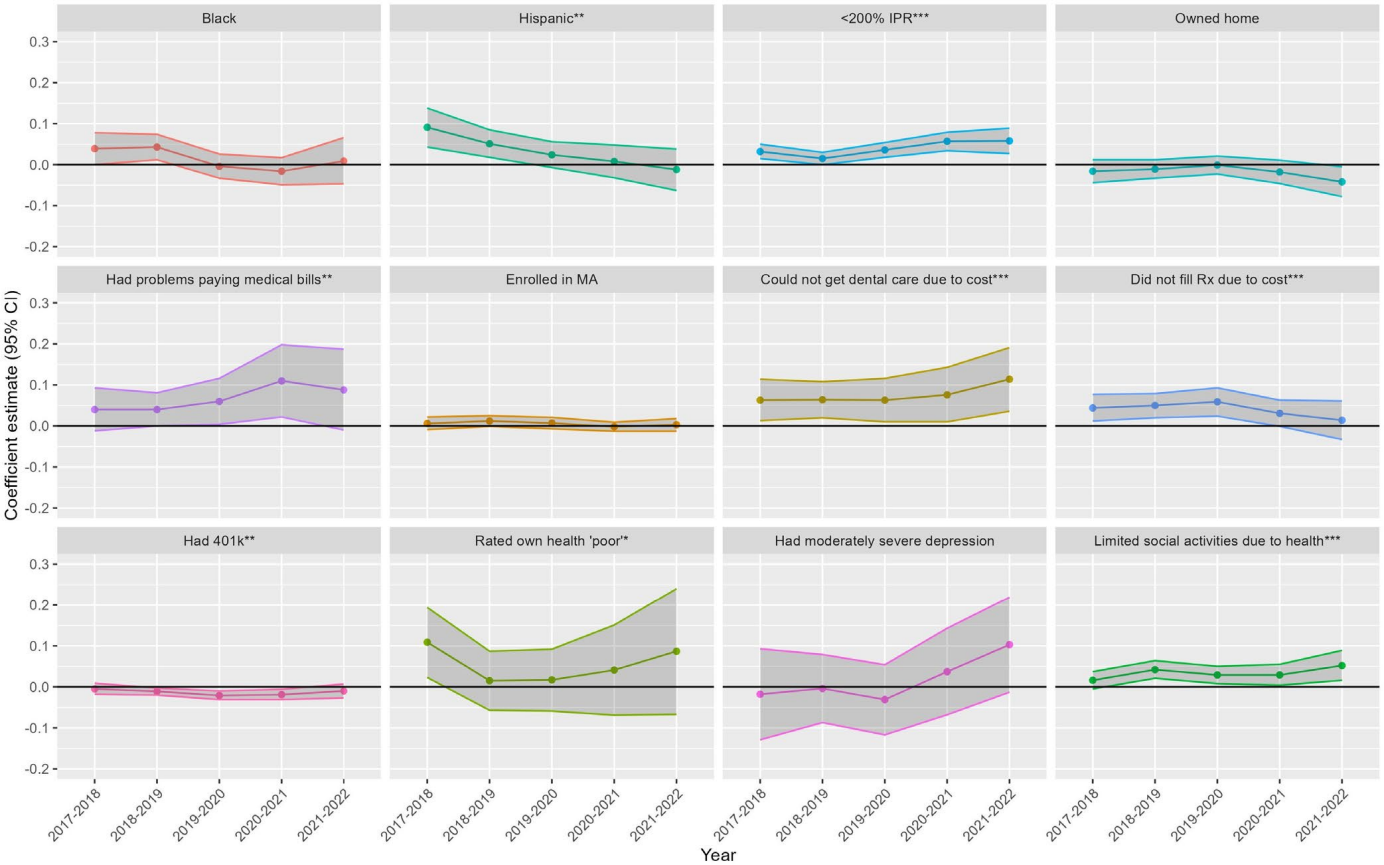
Panel‐specific effects of key predictors of food insecurity across survey Waves, 2017–2018 to 2021–2022. Estimated regression coefficients and 95% confidence intervals for selected demographic, socioeconomic, and health‐related variables across survey years (2017–2018 to 2021–2022). Points represent coefficient estimates, and shaded areas indicate 95% confidence intervals. Panels display results for each variable, including race/ethnicity, income, insurance status, financial strain, access to care, and health outcomes. Statistical significance is denoted as *p < 0.01, **p < 0.05, ***p < 0.001, based on pooled cohort analyses.

Panel‐specific effects (Figure 1) indicated that most coefficients were stable across periods, suggesting little evidence that the COVID‐19 pandemic altered the relative importance of any of the examined covariates. One possible exception was depression, which had little predictive power before 2020 but showed an upward trend in predicting food insecurity among older adults, although this association did not reach statistical significance.

## 4 Discussion

We examined key predictors of food insecurity among Medicare beneficiaries. After adjusting for demographics, socioeconomic status, and health conditions, prior‐year food insecurity was the strongest predictor of subsequent food insecurity. Even after accounting for income level, healthcare‐related financial strain, such as the inability to afford medical care, dental care, or prescriptions, remained a strong predictor. Hispanic ethnicity was the only demographic characteristic that remained significantly associated with food insecurity.

Some findings were consistent with previous studies on the determinants of food insecurity. For example, we confirmed that disposable income, assets, and race and ethnicity were significant predictors of food insecurity [36, 37]. Among beneficiaries aged 65+, we also found that older age was associated with lower rates of food insecurity [21, 38, 39], and that this association persisted after adjusting for underlying health conditions. This pattern may partly reflect selection effects, as individuals with poorer health may be less likely to survive to older ages or if they do, to remain in community‐based dwellings, which are not captured in our sample.

Our study has several key implications. First, among Medicare beneficiaries, socioeconomic factors, including wealth, income, and medical spending, largely explained the unadjusted disparity in food insecurity between Black and non‐Black beneficiaries. This contrasts with prior work suggesting that non‐socioeconomic factors (e.g., structural, behavioral, or institutional factors) may play a larger role [40]. To the extent that those non‐socioeconomic factors contribute to disparities in food security, our findings suggest their effects may operate partly through healthcare‐related financial pathways. It is also possible that Medicare coverage attenuates some disparities observed in the younger Black adult population.

Second, Hispanic Medicare beneficiaries appeared similarly vulnerable to food insecurity as Hispanic working‐age adults, based on comparable effect sizes reported in our and prior studies [38, 39]. This disparity persisted after adjusting for socioeconomic and health differences, and subgroup analyses did not identify a clear explanatory factor. This pattern may reflect unmeasured influences—such as language barriers, unfamiliarity with benefits, health behaviors, or cultural differences—beyond those captured in our data.

Third, the inability to afford dental care emerged as a strong and consistent predictor of food insecurity. Among beneficiaries who were food insecure at baseline, inability to afford dental care was associated with nearly a 13 p.p. higher probability of food insecurity in the following year—more than twice the effect observed in the overall sample. This finding suggests that limited dental coverage under traditional Medicare may intensify financial strain among beneficiaries already vulnerable to food insecurity. Expansion of Medicare dental benefits, including coverage of preventive and restorative dental services, might help mitigate dental care‐related financial strain among Medicare beneficiaries vulnerable to food insecurity [41].

Finally, out‐of‐pocket medical and prescription costs were more strongly associated with food insecurity than specific diagnosed conditions [14]. While individual health conditions were not significant after socioeconomic adjustment, general health indicators remained predictive. This differs from some prior work [14] and may reflect the greater financial vulnerability of Medicare beneficiaries, who often face substantial medical needs on fixed incomes. The frequent association of cost‐related care avoidance and difficulty paying medical bills with food insecurity highlights the ongoing relevance of medical debt [42] and the “treat or eat” tradeoff [43].

### 4.1 Limitations

Although our longitudinal design helps mitigate reverse‐causality concerns inherent in cross‐sectional studies, our results shoudl be interpreted as predictive associations rather than causal effects, as the estimates may be subject to bias from unobserved confounders (e.g., variations in local food access and prices, availability of informal support, or ability to navigate assistance programs). The lagged dependent variable approach assumes prior‐year predictors influence subsequent food insecurity after adjusting for observed covariates. This assumption may be violated by measurement error, such as delayed diagnoses or reporting inaccuracies.

While MCBS allowed us to account for underlying health conditions often omitted from prior research, the survey excludes nursing home residents and does not capture SNAP participation, both of which are relevant to food insecurity. In addition, because the Food Security Survey Module measures household‐level food insecurity, not individual‐level, some misclassification is possible. As this misclassification is likely to be non‐differential (i.e., the measurement error is not systematically related to the outcome variable), this would likely bias estimates toward the null [7].

Future research should further examine the longitudinal relationship between medical spending and food insecurity, the inverse association between age and food insecurity, the drivers of disparities among Black and Hispanic beneficiaries, and the determinants of persistent food insecurity among older adults.

## 5 Conclusions

In this nationally representative longitudinal study of Medicare beneficiaries, prior‐year food insecurity was the strongest predictor of subsequent food insecurity. We observed disparities in food insecurity by race and ethnicity. However, the association for Black beneficiaries was largely explained by socioeconomic factors, whereas Hispanic ethnicity remained predictive even after adjustment for socioeconomic factors and underlying health conditions. Markers of acute financial strain, including difficulty affording dental care or prescriptions, were strong predictors of subsequent food insecurity, independent of income. Incorporating these indicators into screening efforts may improve identification of beneficiaries at risk of food insecurity. Furthermore, linking screening efforts with navigation and benefits enrollment support, alongside broader policy reforms to reduce financial strain, may help mitigate food insecurity and its downstream health consequences among older adults.

## Author Contributions

**David D. Kim:** funding and data acquisition, conceptualization, methodology, drafting, and revising the manuscript. **Charles J. Duncan:** data analysis, visualization, drafting the manuscript. **Elliott Crummer:** data analysis, revising the manuscript.

## Funding

The project was supported by NIH/NIMHD R01MD019094. The sponsor had no role in the design, methods, analysis, or preparation of the manuscript.

## Conflicts of Interest

The authors declare no conflicts of interest.

## Supporting information


**Table S1:** Models of food insecurity for Medicare beneficiaries aged 65+, 2017–2022.
**Table S2:** Linear Probability Model of food insecurity among Medicare beneficiaries aged 65+, 2017–2022, stratified by age.
**Table S3:** Linear Probability Model of food insecurity among Medicare beneficiaries aged 65+, 2017–2022, stratified by food insecurity status in their first observed year (baseline), 2017–2022.
