# Supplementary material for: Food Insecurity Among Medicare Beneficiaries in 2017–2022: A Longitudinal Cohort Study
Source: J Am Geriatr Soc. 2026 Apr 15;74(6):1626–37. doi: 10.1111/jgs.70441 (PMC13266451; doi:10.1111/jgs.70441)
Supplement: Supplementary file 1 — Table S1: Models of food insecurity for Medicare beneficiaries aged 65+, 2017–2022. Table S2: Linear Probability Model of food insecurity among Medicare beneficiaries aged 65+, 2017–2022, stratified by age. Table S3: Linear Probability Model of food insecurity among Medicare beneficiaries aged 65+, 2017–2022, stratified by food insecurity status in their first observed year (baseline), 2017–2022. [file JGS-74-1626-s001.pdf]

Supplementary Table S1. Models of food insecurity for Medicare beneficiaries aged 65+, 2017–2022

	Model 1			Model 2			Model 3		
	LPM	Probit	Logit	LPM	Probit	Logit	LPM	Probit	Logit
Food insecure last year	**0.503***<br>(0.468, 0.539)**	**0.158***<br>(0.149, 0.167)**	**0.142***<br>(0.134, 0.150)**	**0.433***<br>(0.393, 0.474)**	**0.110***<br>(0.101, 0.120)**	**0.100***<br>(0.091, 0.110)**	**0.430***<br>(0.387, 0.472)**	**0.102***<br>(0.092, 0.111)**	**0.093***<br>(0.084, 0.102)**
Demographic characteristics									
Age	**-0.001***<br>(-0.002, -0.001)**	**-0.001***<br>(-0.002, -0.001)**	**-0.001***<br>(-0.002, -0.001)**	**-0.001***<br>(-0.002, -0.001)**	**-0.001***<br>(-0.002, -0.001)**	**-0.001***<br>(-0.002, -0.001)**	**-0.002***<br>(-0.002, -0.001)**	**-0.001***<br>(-0.002, -0.001)**	**-0.001***<br>(-0.002, -0.001)**
Female	**0.008*<br>(0.000, 0.016)**	0.008<br>(0.000, 0.015)	0.008<br>(0.000, 0.016)	0.004<br>(-0.004, 0.012)	0.005<br>(-0.003, 0.014)	0.005<br>(-0.004, 0.014)	0.002<br>(-0.006, 0.010)	0.003<br>(-0.005, 0.011)	0.002<br>(-0.007, 0.012)
Black	**0.049***<br>(0.029, 0.070)**	**0.035***<br>(0.025, 0.046)**	**0.032***<br>(0.022, 0.043)**	0.019<br>(-0.004, 0.042)	**0.014*<br>(0.002, 0.026)**	**0.012*<br>(0.001, 0.024)**	0.018<br>(-0.005, 0.041)	**0.013*<br>(0.001, 0.025)**	0.011<br>(0.000, 0.023)
Hispanic	**0.054***<br>(0.032, 0.075)**	**0.038***<br>(0.027, 0.049)**	**0.035***<br>(0.024, 0.046)**	**0.035**<br>(0.011, 0.059)**	**0.019**<br>(0.007, 0.031)**	**0.020**<br>(0.008, 0.032)**	**0.041**<br>(0.017, 0.066)**	**0.023***<br>(0.012, 0.035)**	**0.023***<br>(0.012, 0.035)**
Married	**-0.036***<br>(-0.044, -0.027)**	**-0.036***<br>(-0.044, -0.028)**	**-0.036***<br>(-0.043, -0.028)**	**-0.019***<br>(-0.028, -0.009)**	**-0.021***<br>(-0.030, -0.013)**	**-0.022***<br>(-0.031, -0.013)**	**-0.016**<br>(-0.026, -0.006)**	**-0.020***<br>(-0.028, -0.011)**	**-0.020***<br>(-0.029, -0.011)**
Household size	**0.006**<br>(0.002, 0.011)**	**0.005***<br>(0.002, 0.008)**	**0.005**<br>(0.002, 0.008)**	0.003<br>(-0.002, 0.009)	0.003<br>(0.000, 0.007)	0.003<br>(-0.001, 0.006)	0.002<br>(-0.004, 0.008)	0.003<br>(-0.001, 0.006)	0.002<br>(-0.002, 0.006)
Socioeconomic characteristics									
Income-poverty ratio ≤ 200%	-	-	-	**0.044***<br>(0.033, 0.055)**	**0.037***<br>(0.029, 0.044)**	**0.038***<br>(0.030, 0.046)**	**0.039***<br>(0.028, 0.050)**	**0.031***<br>(0.023, 0.039)**	**0.032***<br>(0.024, 0.040)**
Owns home	-	-	-	**-0.020*<br>(-0.036, -0.004)**	**-0.011*<br>(-0.020, -0.002)**	-0.009<br>(-0.018, 0.000)	-0.016<br>(-0.032, 0.000)	-0.008<br>(-0.017, 0.002)	-0.006<br>(-0.015, 0.003)
Employed	-	-	-	-0.005<br>(-0.014, 0.004)	-0.002<br>(-0.012, 0.009)	-0.001<br>(-0.012, 0.011)	0.002<br>(-0.007, 0.011)	0.006<br>(-0.004, 0.016)	0.007<br>(-0.003, 0.018)
Less than high school education	-	-	-	0.004<br>(-0.019, 0.027)	0.003<br>(-0.008, 0.015)	0.001<br>(-0.01, 0.012)	-0.003<br>(-0.027, 0.021)	-0.002<br>(-0.014, 0.009)	-0.004<br>(-0.015, 0.008)
Had a problem paying medical bills	-	-	-	**0.059**<br>(0.024, 0.095)**	**0.023***<br>(0.010, 0.036)**	**0.021***<br>(0.009, 0.034)**	**0.052**<br>(0.015, 0.089)**	**0.018**<br>(0.004, 0.031)**	**0.016*<br>(0.004, 0.029)**
Enrolled in Medicare Advantage	-	-	-	-0.002<br>(-0.010, 0.006)	-0.001<br>(-0.008, 0.006)	-0.001<br>(-0.009, 0.007)	0.007<br>(-0.002, 0.015)	0.009<br>(0.000, 0.018)	0.010<br>(0.000, 0.019)
Not enough money to get healthcare	-	-	-	-0.032<br>(-0.135, 0.071)	-0.007<br>(-0.046, 0.033)	-0.008<br>(-0.048, 0.031)	-0.047<br>(-0.16, 0.065)	-0.012<br>(-0.052, 0.027)	-0.014<br>(-0.054, 0.026)
Unable to get dental care due to cost	-	-	-	**0.070***<br>(0.038, 0.103)**	**0.026***<br>(0.014, 0.039)**	**0.025***<br>(0.013, 0.037)**	**0.064***<br>(0.031, 0.097)**	**0.023***<br>(0.011, 0.035)**	**0.023***<br>(0.012, 0.034)**
Often/sometimes does not fill Rx due to cost	-	-	-	**0.056***<br>(0.036, 0.076)**	**0.037***<br>(0.028, 0.046)**	**0.037***<br>(0.028, 0.047)**	**0.044***<br>(0.022, 0.065)**	**0.028***<br>(0.018, 0.037)**	**0.029***<br>(0.020, 0.039)**
Has 401k	-	-	-	**-0.012**<br>(-0.02, -0.005)**	**-0.026***<br>(-0.037, -0.015)**	**-0.029***<br>(-0.042, -0.017)**	**-0.011**<br>(-0.019, -0.003)**	**-0.024***<br>(-0.035, -0.013)**	**-0.028***<br>(-0.040, -0.016)**
Health conditions									
General health felt to be "poor"	-	-	-	-	-	-	**0.065*<br>(0.005, 0.124)**	0.015<br>(-0.005, 0.036)	0.015<br>(-0.005, 0.035)
Has difficulty walking	-	-	-	-	-	-	0.009<br>(-0.004, 0.022)	0.007<br>(-0.001, 0.015)	0.007<br>(-0.002, 0.015)
Has cognitive difficulties	-	-	-	-	-	-	0.001<br>(-0.025, 0.027)	0.000<br>(-0.013, 0.013)	-0.001<br>(-0.014, 0.012)
Has at least moderately severe depression	-	-	-	-	-	-	0.000<br>(-0.061, 0.061)	-0.005<br>(-0.030, 0.020)	-0.008<br>(-0.030, 0.015)
Has clinical anxiety	-	-	-	-	-	-	0.000<br>(-0.018, 0.019)	0.002<br>(-0.009, 0.013)	0.001<br>(-0.010, 0.011)
Limited social activities due to health	-	-	-	-	-	-	**0.031***<br>(0.015, 0.046)**	**0.021***<br>(0.011, 0.030)**	**0.021***<br>(0.011, 0.030)**

Has smoked 100+ cigarettes	-	-	-	-	-	-	0.002 (-0.006, 0.010)	0.005 (-0.003, 0.012)	0.004 (-0.004, 0.013)
Has Alzheimer's	-	-	-	-	-	-	-0.027 (-0.057, 0.003)	-0.031 (-0.063, 0.002)	-0.030 (-0.064, 0.004)
Has CKD	-	-	-	-	-	-	0.010 (-0.006, 0.026)	0.010 (-0.003, 0.023)	0.010 (-0.003, 0.024)
Has COPD	-	-	-	-	-	-	-0.014 (-0.037, 0.009)	-0.010 (-0.027, 0.008)	-0.012 (-0.029, 0.006)
Has cardiovascular disease	-	-	-	-	-	-	0.003 (-0.010, 0.017)	0.002 (-0.011, 0.015)	0.003 (-0.011, 0.017)
Has diabetes	-	-	-	-	-	-	0.006 (-0.007, 0.020)	0.006 (-0.005, 0.017)	0.006 (-0.006, 0.018)
Has rheumatoid/osteoarthritis	-	-	-	-	-	-	0.008 (-0.002, 0.018)	0.010 (0.000, 0.019)	0.009 (-0.001, 0.020)
N (observations)	16412	16412	16412	14970	14970	14970	13769	13769	13769
N (individuals)	11749	11749	11749	10796	10796	10796	10059	10059	10059
AIC	-1876	5798	5828	-3100	4685	4731	-3812	4085	4127
BIC	830	5789	5821	807	4756	4803	847	4265	4309
Log.Lik	-376	-2856	-2872	-317	-2291	-2315	-276	-1985	-2007

Note: Boldface indicates statistical significance (*p < 0.05, **p < 0.01, *** p < 0.001). LPM = linear probability model; CKD = chronic kidney disease; COPD = chronic obstructive pulmonary disease. 95% confidence intervals in parentheses. All estimates account for complex survey design.

Supplementary Table S2. Linear Probability Model of food insecurity among Medicare beneficiaries aged 65+, 2017-2022, stratified by age

	Age 65-74	Age 75+
Food insecure in prior year	**0.451***<br>(0.397, 0.505)**	**0.368***<br>(0.310, 0.427)**
Demographic characteristics		
Age	**-0.002*<br>(-0.004, 0.000)**	**-0.002**<br>(-0.003, -0.001)**
Female [vs Male]	0.005<br>(-0.005, 0.015)	-0.002<br>(-0.012, 0.008)
Black [vs White or other]	0.015<br>(-0.017, 0.047)	0.023<br>(-0.007, 0.052)
Hispanic [vs White or other]	**0.041*<br>(0.009, 0.074)**	**0.031*<br>(0.002, 0.060)**
Married [vs Not married]	-0.013<br>(-0.028, 0.001)	**-0.023***<br>(-0.034, -0.011)**
Household size	0.001<br>(-0.007, 0.009)	0.004<br>(-0.004, 0.011)
Socioeconomic characteristics		
Income-poverty ratio<br>≤ 200%	**0.040***<br>(0.024, 0.057)**	**0.036***<br>(0.022, 0.049)**
Owns home	-0.020<br>(-0.043, 0.002)	-0.010<br>(-0.029, 0.010)
Employed	0.004<br>(-0.007, 0.015)	0.005<br>(-0.010, 0.020)
Less than high school education	0.007<br>(-0.029, 0.044)	-0.014<br>(-0.039, 0.010)
Had a problem paying medical bills	0.045<br>(-0.001, 0.091)	**0.072*<br>(0.011, 0.133)**
Enrolled in Medicare Advantage	0.008<br>(-0.003, 0.019)	0.000<br>(-0.011, 0.012)
Not enough money to get healthcare	**-0.122*<br>(-0.240, -0.004)**	0.210<br>(-0.014, 0.433)
Unable to get dental care due to cost	**0.054**<br>(0.015, 0.093)**	**0.085**<br>(0.035, 0.134)**
Often/sometimes does not fill Rx due to cost	**0.054***<br>(0.027, 0.081)**	0.016<br>(-0.012, 0.044)
Has 401k	**-0.012*<br>(-0.022, -0.002)**	**-0.009*<br>(-0.017, 0.000)**
Health conditions		
General health felt to be poor	0.065<br>(-0.010, 0.139)	0.050<br>(-0.025, 0.124)
Has difficulty walking	0.008<br>(-0.015, 0.032)	**0.013*<br>(0.002, 0.025)**
Has cognitive difficulties	0.014<br>(-0.027, 0.055)	-0.019<br>(-0.042, 0.005)
Has at least moderately severe depression	-0.009<br>(-0.090, 0.072)	0.021<br>(-0.066, 0.108)
Has clinical anxiety	-0.009<br>(-0.034, 0.017)	0.016<br>(-0.012, 0.045)
Limited social activities due to health	**0.045***<br>(0.021, 0.069)**	0.007<br>(-0.007, 0.022)
Has smoked 100+ cigarettes	0.002<br>(-0.008, 0.012)	0.004<br>(-0.005, 0.014)
Has Alzheimer's	-0.036<br>(-0.097, 0.025)	-0.01<br>(-0.044, 0.025)
Has CKD	0.016<br>(-0.010, 0.043)	0.004<br>(-0.011, 0.019)
Has COPD	-0.024<br>(-0.054, 0.007)	-0.002<br>(-0.032, 0.027)
Has cardiovascular disease	0.006<br>(-0.013, 0.025)	-0.002<br>(-0.015, 0.011)
Has diabetes	-0.004<br>(-0.023, 0.014)	**0.020*<br>(0.004, 0.036)**
Has rheumatoid/osteoarthritis	0.014<br>(-0.002, 0.030)	-0.005<br>(-0.013, 0.004)
N (observations)	6,587	7,179
N (individuals)	4,768	5,418

Note: Boldface indicates statistical significance (*p < 0.05, **p < 0.01, *** p < 0.001). CKD = chronic kidney disease; COPD = chronic obstructive pulmonary disease. 95% confidence intervals in parentheses.

Supplementary Table S3. Linear Probability Model of food insecurity among Medicare beneficiaries aged 65+, 2017-2022, stratified by food insecurity status in their first observed year (baseline), 2017-2022

	Food Secure at Baseline	Food Insecure At Baseline
Food insecure in prior year	**0.288*** (0.163, 0.413)**	**0.239*** (0.133, 0.344)**
Demographic characteristics		
Age	**-0.001*** (-0.002, -0.001)**	**-0.007* (-0.012, -0.002)**
Female [vs Male]	0.002 (-0.004, 0.008)	0.021 (-0.070, 0.112)
Black [vs White or other]	**0.024* (0.001, 0.046)**	-0.019 (-0.108, 0.070)
Hispanic [vs White or other]	**0.039** (0.013, 0.066)**	0.075 (-0.016, 0.166)
Married [vs Not married]	**-0.013** (-0.022, -0.003)**	-0.089 (-0.177, 0.000)
Household size	0.003 (-0.003, 0.008)	-0.003 (-0.032, 0.026)
Socioeconomic characteristics		
Income-poverty ratio ≤ 200%	**0.034*** (0.023, 0.044)**	0.058 (-0.047, 0.163)
Owns home	**-0.017* (-0.031, -0.002)**	-0.002 (-0.077, 0.074)
Employed	0.001 (-0.007, 0.010)	0.042 (-0.073, 0.157)
Less than high school education	0.008 (-0.013, 0.028)	-0.059 (-0.149, 0.031)
Had a problem paying medical bills	**0.042* (0.007, 0.078)**	0.055 (-0.032, 0.142)
Enrolled in Medicare Advantage	**0.008* (0.002, 0.014)**	-0.021 (-0.122, 0.081)
Not enough money to get healthcare	-0.014 (-0.108, 0.079)	-0.163 (-0.422, 0.096)
Unable to get dental care due to cost	0.024 (-0.015, 0.063)	**0.129* (0.029, 0.229)**
Often/sometimes does not fill Rx due to cost	**0.021* (0.004, 0.037)**	**0.194*** (0.093, 0.294)**
Has 401k	**-0.010** (-0.017, -0.004)**	-0.064 (-0.187, 0.060)
Health conditions		
General health felt to be poor	0.052 (-0.016, 0.120)	0.090 (-0.063, 0.243)
Has difficulty walking	0.006 (-0.005, 0.017)	0.046 (-0.038, 0.131)
Has cognitive difficulties	0.015 (-0.008, 0.038)	-0.078 (-0.182, 0.026)
Has at least moderately severe depression	0.017 (-0.048, 0.083)	-0.056 (-0.193, 0.081)
Has clinical anxiety	0.001 (-0.015, 0.018)	-0.006 (-0.102, 0.089)
Limited social activities due to health	**0.021** (0.009, 0.033)**	**0.090* (0.005, 0.175)**
Has smoked 100+ cigarettes	0.005 (-0.001, 0.012)	-0.030 (-0.108, 0.049)
Has Alzheimer's	-0.018 (-0.041, 0.005)	-0.128 (-0.459, 0.202)
Has CKD	0.005 (-0.010, 0.019)	0.083 (-0.040, 0.206)
Has COPD	-0.007 (-0.027, 0.013)	-0.095 (-0.271, 0.081)
Has cardiovascular disease	0.004 (-0.007, 0.015)	-0.034 (-0.169, 0.101)
Has diabetes	0.007 (-0.004, 0.017)	-0.010 (-0.127, 0.108)
Has rheumatoid/osteoarthritis	0.006 (-0.002, 0.014)	0.050 (-0.060, 0.160)
N (observations)	12,872	893
N (individuals)	9,374	682

Note: Boldface indicates statistical significance (*p < 0.05, **p < 0.01, *** p < 0.001). CKD = chronic kidney disease; COPD = chronic obstructive pulmonary disease. 95% confidence intervals in parentheses.
